# Interleukin-33 and soluble suppression of tumorigenicity 2 in scleroderma cardiac involvement

**DOI:** 10.1007/s10238-022-00864-7

**Published:** 2022-07-25

**Authors:** Francesco Iannazzo, Chiara Pellicano, Amalia Colalillo, Cesarina Ramaccini, Antonella Romaniello, Antonietta Gigante, Edoardo Rosato

**Affiliations:** 1 Department of Translational and Precision Medicine – Scleroderma Unit, Sapienza University of Rome, Viale dell’Università 37, 00185 Rome, Italy; 2 Division of Cardiology, Sant’Andrea Hospital, Rome, Italy

**Keywords:** Systemic sclerosis, Echocardiography, Heart rate variability, IL-33, sST2

## Abstract

Interleukin (IL)-33 is part of the IL-1 family of cytokines and soluble suppression of tumorigenicity 2 (sST2) is part of the family of IL-1 receptors. In systemic sclerosis (SSc), IL-33 and sST2 are involved in cardiac manifestations such as diastolic dysfunction (DD), autonomic dysfunction (AD) and right ventricular–pulmonary arterial coupling assessed by tricuspid annular plane systolic excursion (TAPSE)/systolic pulmonary artery pressure (sPAP). Serum levels of IL33 and sST2 were assessed in 50 SSc patients and 14 healthy controls (HC). Clinical assessment, echocardiography and heart rate variability (HRV) analysis were performed in SSc patients. Serum levels of IL-33 and sST2 were significantly higher in SSc patients than HC. A linear positive correlation between modified Rodnan skin score and IL33 was observed. Serum values of sST2 were higher in SSc patients with DD than in patients without DD [15403 pg/ml (12,208–19,941) vs 8556 pg/ml (6820–11,036), *p* < 0.001]. sST2 showed a negative correlation with standard deviation of normal-to-normal RR intervals (SDNN) (*r* = − 0.281, *p* < 0.05) and positive correlation with low frequency/high frequency (LF/HF) (*r* = 0,349, *p* < 0.01). Negative linear correlation exists between sST2 and TAPSE/sPAP (*r* = − 0.398, *p* < 0.01). Serum levels of IL-33 and sST2 are higher in SSc patients than HC. Serum levels of sST2 are a potential marker of DD, AD and right ventricular–pulmonary arterial coupling.

## Introduction

Systemic Sclerosis (SSc) is an autoimmune disease characterized by microvascular damage, immune system dysregulation and fibrosis of skin and internal organs.

Several forms of SSc heart involvement have been reported such as cardiac Raynaud's phenomenon (RP), myocardial fibrosis, left ventricular (LV) systolic dysfunction, LV diastolic dysfunction, pericardial disease and conduction abnormalities [1].

Asymptomatic involvement of the heart was found in 70% of SSc patients [2]. The most common manifestation of cardiac involvement was diastolic dysfunction (DD), conversely LV systolic dysfunction was found in 3–5% SSc patients. DD is independently associated with an increased risk of mortality in patients with SSc [3].

Echocardiography is used to evaluate LV and right ventricular (RV) systolic and diastolic function.

Tissue Doppler imaging (TDI) is a recently developed ultrasonographic technique that enables quantitative analysis of global and regional myocardial LV and RV function. DD assessment includes 4 measures: left atrial volume index (LAVI), septal and lateral early relaxation velocity on tissue Doppler (e’), early filling velocity on transmitral Doppler/early relaxation velocity on tissue Doppler (E/e’) ratio, tricuspid regurgitation (TR) systolic jet velocity [4]. Tricuspid annular plane systolic excursion (TAPSE)/systolic pulmonary artery pressure (sPAP) ratio was used as a coupling index between RV and pulmonary artery [5].

Heart Rate Variability (HRV) analysis can identify autonomic dysfunction (AD) in asymptomatic SSc patients. Standard deviation of normal-to-normal RR intervals (SDNN) and low frequency/high frequency (LF/HF) ratio are the most used parameters to assess AD [6]. AD is an early feature of SSc and it is associated with increased cardiovascular risk. AD was reported in SSc patients with reduced myocardial vasodilatory response after cold test, increased intrarenal arterial stiffness and reduced exercise capacity [7–10].

Interleukin (IL)-33 is member of the IL-1 family of cytokines. Receptor of IL-33, suppression of tumorigenicity 2 (ST2), exists in a transmembrane (ST2L) and soluble form (sST2). The interaction between IL-33 and ST2L induces release pro-inflammatory cytokines such as IL-4, IL-5 and IL-13.

The transmembrane form of ST2 enables IL-33’s signaling activity, whilst sST2 acts as a decoy receptor binding IL-33, to dampen its effects. IL-33 plays a key role in the initiation and progression of numerous fibrotic and/or inflammatory diseases such as pulmonary fibrosis, asthma, rheumatoid arthritis and inflammatory bowel diseases [11]. In preclinical studies, IL-33 showed cardioprotective effects by reducing myocardial fibrosis and hypertrophy [12].

Brain Natriuretic Peptides (BNPs) are widely used as a biomarker of acute and chronic cardiac conditions. In some small studies, sST2 demonstrated a higher predictive value of mortality in patients with acute heart failure, chronic heart failure and myocardial infarction [13].

Aim of the study is to evaluate the serum level of IL-33 and sST2 and to assess the relationship between serum level of IL-33 or sST2 and diastolic dysfunction, autonomic dysfunction and right ventricle impairment in SSc patients.

## Materials and methods

### Subjects

In this study, we enrolled 50 SSc patients [45 females, median age 57 (IQR 51–63) years] who fulfilled 2013 revised ACR/EULAR criteria [14]. 29 patients had limited cutaneous SSc (lcSSc) and 21 diffuse cutaneous (dcSSc) SSc according to LeRoy et al. [15].

Exclusion criteria were unstable angina, heart failure, arrhythmias, valvular heart disease, uncontrolled arterial hypertension, hypertrophic cardiomyopathy, diabetes, pulmonary diseases not related to SSc, pregnancy and breastfeeding. We also excluded SSc patients treated with Angiotensin converting enzyme (ACE) inhibitors, beta blockers and anti-arrhythmic drugs, angiotensin II receptor blockers, mineralocorticoid receptor antagonists.

Fourteen healthy controls (HC) were also enrolled [12 females; median age 52 (IQR 47–65) years].

The subjects' written consents were obtained according to the Declaration of Helsinki and the study was conducted in agreement to local ethics committee directives.

### Serum level of IL33 and sST2

Serum level of IL33/ (pg/ml) were measured by ELISA Kit (MyBioSource, San Diego, USA). Serum level of sST2 (pg/ml) were measured by ELISA kit (Abcam, Cambridge, UK). Blood samples were collected after a 12-h overnight fast.

### Echocardiography

Echocardiograms were performed with the General Electric Vivid S5 apparatus (GE Medical Systems, Israel Ltd.). All patients were studied in left lateral decubitus. DD was evaluated in according of international guidelines of 2016 [4]. LV diameters, wall thickness, ejection fraction (EF), RV diameter, tricuspidal annular plane systolic excursion, left and right atrium area and volume, sPAP were measured.

#### HRV

All subjects underwent 24 h ambulatory 3‐channel ECG Holter recording (Lifecard CF, Spacelabs Healthcare, Snoqualmie,WA, USA). Autonomic nervous activity was assessed by HRV in time and frequency domain according to the recommendation of the European Society of Cardiology and the North American Society of Pacing and Electrophysiology [16].

Spectral estimates of normal‐to‐normal RR intervals (NN) were obtained from stationary intervals free of ectopic beats and technical artifacts. The following parameters were computed in time domain analysis: SDNN which captures total HRV and reflects circadian heart rhythm, and the square root of the mean of the sum of the squares of differences between adjacent NN intervals (RMSSD), which correlates with the parasympathetic modulation of HR In the frequency domain Fast Fourier Transform was used to obtain power spectral estimates of HRV. Total power in the frequency range (0–0.40 Hz) was divided into low frequency (LF 0.04–0.15 Hz, modulated mainly by sympathetic system) and high frequency (HF 0.15–0.40 Hz, modulated by parasympathetic system). HF is an indicator of parasympathetic activity, conversely LF is an indicator of sympathetic activity. Data analyses were performed with Cardionavigator plus software package (Spacelabs Healthcare, Snoqualmie, WA, USA).

### Clinical assessment

Modified Rodnan skin score (mRss), disease activity index (DAI) and disease severity scale (DSS) were performed in SSc patients [17, 18]. Nailfold videocapillaroscopy (NVC) was performed with a videocapillaroscope (Pinnacle Studio Version 8) equipped with a 500 × optical probe. According to Cutolo et al. [19] patterns identified within the “SSc pattern” include: early, active and late. SSc patients were evaluated to estimate ILD by pulmonary function tests (PFTs) according to the standards recommended by the American/European Respiratory Society [20].

### Statistical analysis

All results are expressed as mean ± SD or median and IQR, as appropriate. SPSS version 25.0 software was used for the statistical analysis. The Shapiro–Wilk test was used to evaluate normal distribution of data. Group comparisons were made by Student’s unpaired 2-tailed *t*-test or Mann–Whitney test, as appropriate. Pearson product-moment correlation coefficient or Spearman's rank correlation coefficient, as appropriate, was used to test for an association between numerical variables. The chi-square test or Fisher’s exact test, as appropriate, was used to compare categorical variables.

Multiple regression analysis was used to evaluate the correlation between dependent variable (sST2) and continuous independent variables (SDNN, LF/HF, TAPSE/sPAP, age). We included in the multiple regression analysis only the variables that had reached significance in the linear correlation analysis. *P* values < 0,05 were considered significant.

## Results

Table 1 shows epidemiological and clinical features of SSc patients.

**Table 1:** Demographic and clinical features of systemic sclerosis patients

Age, years—median (IQR)	57 (51–63)
Female gender—n (%)	45 (90)
Disease duration, years – median (IQR)	11 (7–17)
IcSSc/dcSSc, n	29/21
SSc-specific autoantibodies, n (%)	
Scl70	22 (44)
Anticentromere	21 (42)
Anti-RNApolimerase II	2 (4)
None	5 (10)
Nailfold video capillaroscopy, n (%)	
Early	8 (16)
Active	14 (28)
Late	28 (56)
Ulcers history, n (%)	27 (50)
mRss, median (IQR)	11 (8–20)
DAI, median (IQR)	1.50 (0.92–3.75)
DSS, median (IQR)	4 (3–6)
Diastolic dysfunction, n (%)	8 (16)
FVC, % predicted	99 (87–112)
DLCO, % predicted	69.5 (58–78)
sPAP, mmHg	31 (27–41)
LVEF, %	60 (56–63)

IQR: interquartile range; IcSSc: limited cutaneous systemic sclerosis; dcSSc: diffuse cutaneous systemic sclerosis; Scl70: Antitopoisomerase I antibodies; mRss: modified Rodnan skin score, DAI: disease activity index, DSS: disease severity scale; FVC: Forced Vitality capacity; DLCO: diffusing capacity for carbon monoxide; sPAP: systolic Pulmonary Arterial Pressure; LVEF: Left Ventricular Ejection Fraction

### Serum level of IL-33 and sST2

Serum levels of IL-33 were higher in SSc patients than HC [98 pg/ml (70–152) vs 55 pg/ml (28–92), *p* < 0.01]. Serum values of sST2 were higher in SSc patients than HC [9115 pg/ml (6854–12,696) vs 7031 (4862–8269), *p* < 0.05]. In SSc patients, no correlation between serum levels of IL-33 and sST2 was observed. A linear positive correlation between mRSS and IL33 (*r* = 0,270, *p* < 0.05) was observed, while no correlation was found between mRss and sST2. No correlation was observed between age and IL-33 (*r* = − 0.035, *p* > 0.05) and sST2 (*r* = 0.22, *p* > 0.05). No correlation was observed between Forced Vitality capacity (FVC) and IL-33 (*r* = 0.03, *p* > 0.05) or sST2 (*r* = − 0.03, *p* > 0.05). Diffusing capacity for carbon monoxide (DLCO) showed a weak negative correlation with sST2 (*r* = − 0.32, *p* < 0.05). No correlation was observed between DLCO and IL-33 (*r* = − 0.06, *p* > 0.05). No correlation was found between IL-33 or sST2 and disease features (DAI, DSS, disease duration).

### Diastolic dysfunction

**Fig. 1:**
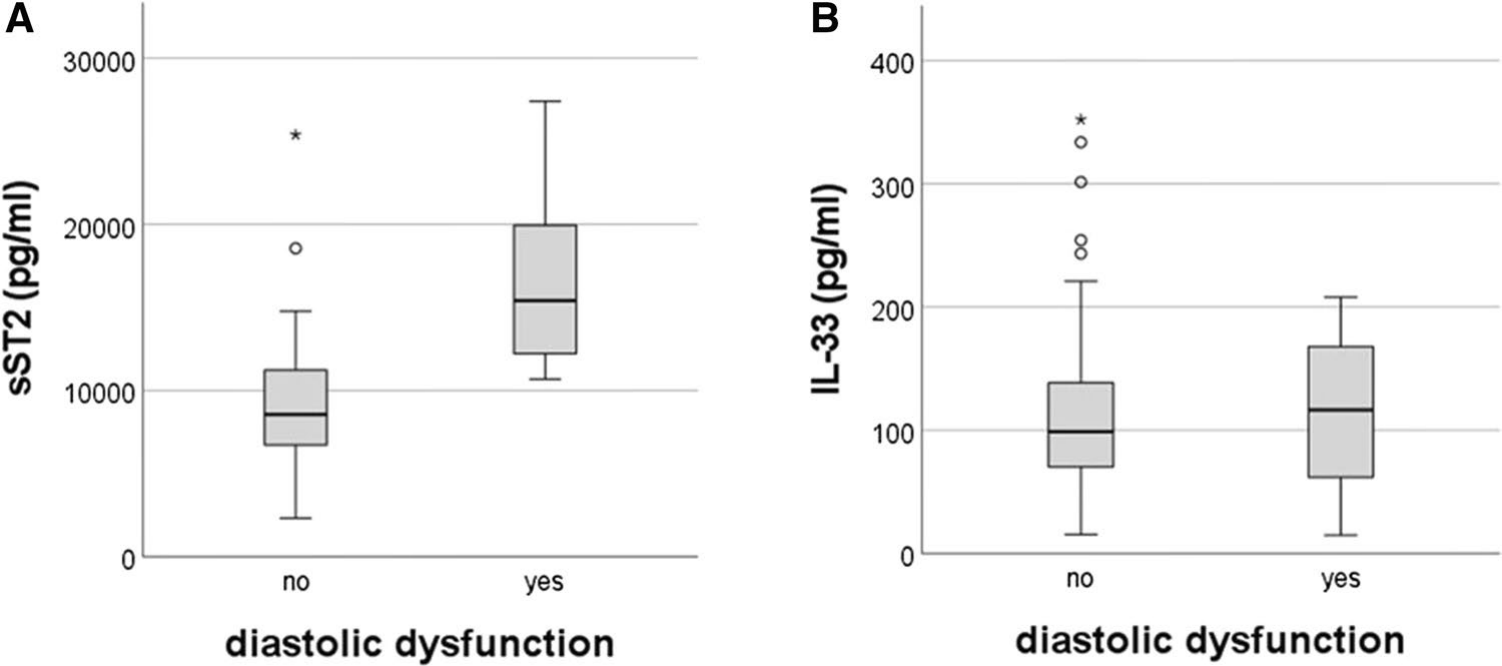
**A** Serum level of soluble suppression of tumorigenicity 2 (sST2) in systemic sclerosis patients with and without diastolic dysfunction; **B** Serum level of Interleukin-33 (IL-33) in systemic sclerosis patients with and without diastolic dysfunction

DD was present in 8 (16%) SSc patients. Serum values of sST2 were higher in SSc patients with DD than in patients without DD [15403 pg/ml (12,208–19,941) vs 8556 pg/ml (6820–11,036), *p* < 0.001] (Fig. 1A). No significant differences of serum level of IL-33 were observed in SSc patients with or without DD [116 pg/ml (61–167) vs 98 pg/ml (70–138), *p* > 0.05] (Fig. 1B). DAI score [4 (1.84–4.46) vs 1.5 (0.84–2.59), *p* < 0.01] and DSS score [6 (5–9) vs 4 (3–6), *p* < 0.05] were higher in SSc with DD than SSc without DD. No significant differences of mRss, age, disease duration were observed in SSc patients with or without DD (Table 2).

**Table 2:** Difference of serological markers and clinical features in systemic sclerosis patients with or without diastolic dysfunction

	Diastolic dysfunction	No diastolic dysfunction	p value
sST2, (pg/ml)	15,403 pg/ml (12,208–19,941)	8556 pg/ml (6720–11,224)	*p* < 0.001
IL-33, (pg/ml)	116 pg/ml (61–167)	98 pg/ml (70–138)	*p* > 0.05
SDNN	119 ms (107–127)	129 ms (120–143)	*p* < 0.05
RMSSD	29 ms (28–38)	37 ms (32–45)	*p* > 0.05
LF	680 (305–2189)	751 (511–1317)	*p* > 0.05
HF	374 (299–3164)	467 (299–890)	*p* > 0.05
LF/HF	2.51 (2.49–2.65)	2.3 (2.13–2.45)	*p* < 0.05
DAI	4 (1.84–4.46)	1.5 (0.84–2.59)	*p* < 0.01
DSS	6 (5–9)	4 (3–6)	*p* < 0.05
mRss	11 (7–18)	12 (8–20)	*p* > 0.05
Disease duration, years	19 (9–23)	11 (5–16)	*p* > 0.05
Age, years	66 (56–72)	57 (48–62)	*p* > 0.05
FVC, % predicted	96.3 (72–106)	99.5 (88–115)	*p* > 0.05
DLCO, % predicted	43.5 (36.5–57)	73 (63–79)	*p* < 0.01

IL-33: Interleukin-33; sST2: soluble suppression of tumorigenicity 2; SDNN: standard deviation of normal-to-normal RR intervals; RMSSD: mean of the sum of the squares of differences between adjacent NN intervals; LF: low frequency; HF: high frequency; LF/HF: low frequency/high frequency ratio; DAI: disease activity index; DSS: disease severity scale; mRss: modified Rodnan skin score; FVC: Forced Vitality capacity; DLCO: diffusing capacity for carbon monoxide

### HRV analysis

**Fig. 2:**
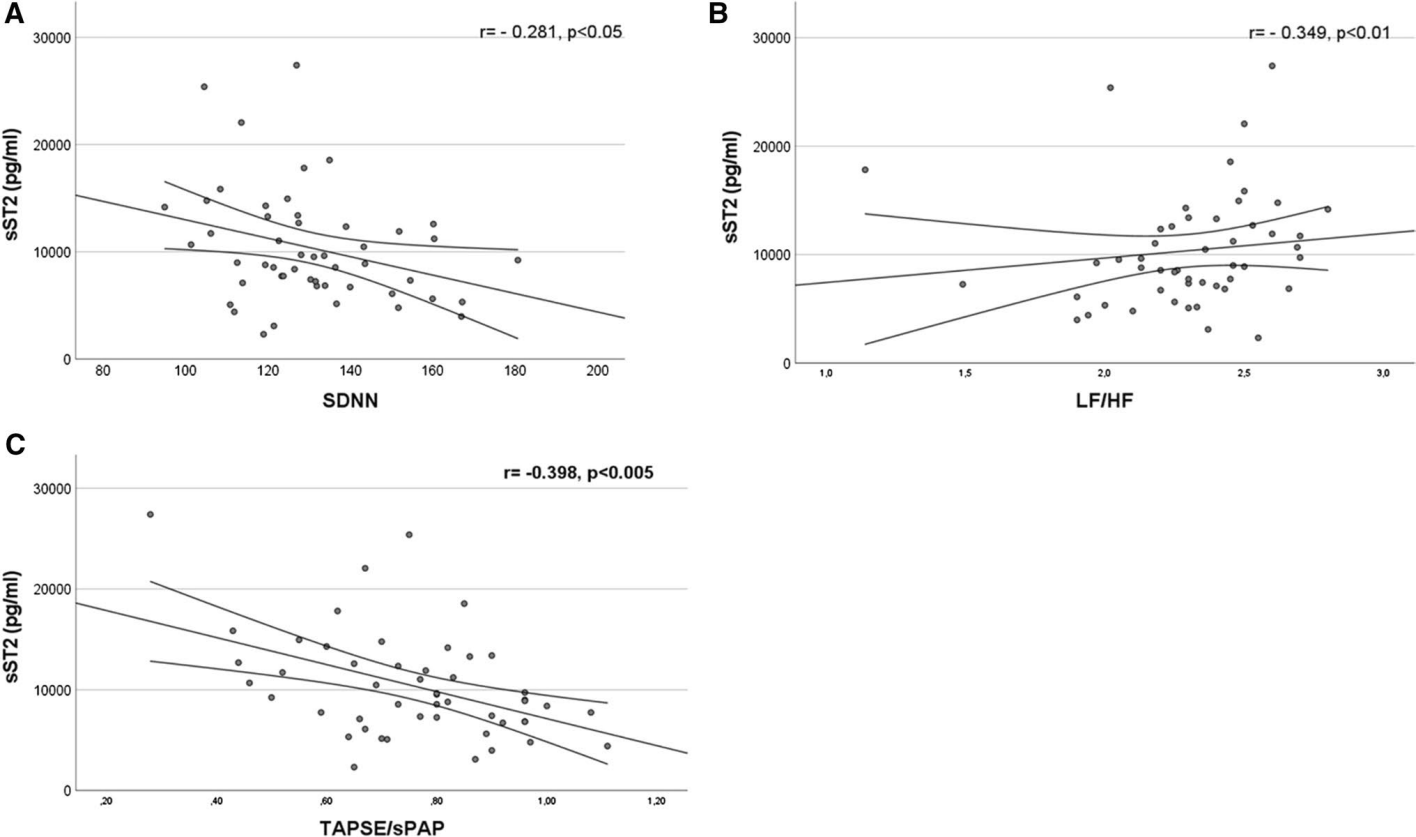
Linear correlation between sST2 and SDNN, sST2 and LF/HF, sST2 and TAPSE/sPAP

Median value of SDNN is 127.8 ms (119.4–140) and median value of LF/HF is 2.32 (2.18–2.50). SDNN showed negative weak correlation with sST2 serum level (*r* = − 0,281, *p* < 0.05) (Fig. 2A), DAI (*r* = − 0.307, *p* < 0.05) and DSS (*r* = − 0.290, *p* < 0.05). No correlation was observed between SDNN and IL-33, mRss, age and disease duration. In multiple regression analysis, SDNN showed a weak correlation with sST2 (β coefficient − 0.306, *p* < 0.05), conversely no correlation was present between SDNN and DAI (β coefficient − 0.172, *p* > 0.05) and DSS (β coefficient − 0.247, *p* > 0.05).

LF/HF showed a weak positive correlation with sST2 (*r* = 0,349, *p* < 0.01) (Fig. 2B). No correlation was observed between LF/HF and IL-33, mRss, DAI, DSS, age and disease duration.

SDNN values were significantly lower in patients with DD than without DD [119 ms (107–127) vs 129 ms (120–143), *p* < 0.05], conversely LF/HF values were higher in patients with DD than patients without DD [2.51 (2.49–2.65) vs 2.3 (2.13–2.43), *p* < 0.05] (Table 2).

### Right ventricular function

Median value of TAPSE/sPAP ratio was 0.78 (IQR 0.65–0.90). Negative linear correlation exists between TAPSE/sPAP and sST2 (*r* = − 0.398, *p* < 0.01) (Fig. 2C), DAI (*r* = − 0.321, *p* < 0.05) and DSS (*r* = − 0.349, *p* < 0.01). No correlation was observed between TAPSE/sPAP and IL-33, mRss, SDNN and LF/HF.

In multiple regression analysis, TAPSE/sPAP ratio showed a significant correlation with sST2 [β coefficient − 0.316, *p* < 0.05], age [β coefficient − 0.329, *p* < 0.01] and DSS [β coefficient − 0.311, *p* < 0.05]. No correlation was present between TAPSE/sPAP ratio and DAI.

## Discussion

In this study, serum levels of IL-33 and sST2 are higher in SSc patients than HC. Serum level of IL-33 shows a weak positive correlation with mRss, conversely serum level of sST2 does not show correlation with mRss. DAI, DSS and sST2 were higher in SSc patients with DD than in SSc patients without SSc. No differences of serum level of IL-33 were observed between SSc patients with or without DD. SDNN showed weak negative correlation with sST2 serum level and weak positive correlation with LF/HF. In multiple regression analysis, only sST2 showed a correlation with SDNN. Negative linear correlation exists between TAPSE/sPAP and sST2, conversely no correlation exists between TAPSE/sPAP and IL-33.

Our results demonstrated that IL-33 is increased in SSc patients and it shows a weak positive correlation with mRss. Previous studies demonstrated that IL-33 is an emerging pro-fibrotic cytokine in SSc fibrosis of lung and skin. The role of sST2 in the pathogenesis of SSc fibrosis is unknown [21]. In our study, sST2 is higher in SSc patients than HC, but no correlation was found with mRss. Wagner et al. demonstrated that sST2 is higher in lcSSc after 9 years of disease and sST2 serum levels were lowered by prostacyclin treatment. Serum sST2 is a biomarker for progressive vascular fibrosis [22]. Serum sST2 levels in patients with progressive disease were significantly elevated compared with patients with stable disease [23].

In this study, DD is present in 8 (16%) patients. sST2 was significantly higher in SSc patients with DD than in SSc patients without DD, conversely IL-33 does not show significant differences in SSc patients with and without DD. In SSc, the DD is due to myocardial fibrosis. The role of IL-33 and sST2 in myocardial damage is controversial. The transmembrane form of ST2 enables IL-33’s signaling activity with cardioprotective effects, whilst sST2 acts as a decoy receptor binding IL-33, to dampen its effects [24]. The binding of sST2 to IL-33 has been associated with myocardial hypertrophy and fibrosis by blocking. Previous meta-analyses have shown that sST2 has diagnostic value for heart failure and is prognostic for all-cause mortality in heart failure, coronary artery disease and following aortic valve replacement [25]. In systemic lupus erythematosus patients, sST2 is a marker of disease activity and DD [26]. We can suppose that IL-33 and sST2 may play a role in the pathogenesis of SSc DD, but these preliminary data need to be confirmed by large studies.

FVC is the most used parameter to evaluate ILD in SSc, conversely DLCO can be both a marker of ILD and pulmonary vasculopathy. DLCO may be reduced in patients with pulmonary arterial hypertension without signs of ILD. In this study, we have seen that DLCO is reduced in SSc patients with DD. We can suppose that DLCO is reduced by pulmonary hypertension due to left heart disease such as DD.

In this study, we demonstrated that SDNN showed a weak negative correlation with sST2 serum level and positive correlation with LF/HF. No correlation was found between IL-33 and SDNN or LF/HF. SDNN and LF/HF ratio are the most used parameters of HRV to assess AD. AD is an early feature of SSc and it could be responsible for the reduced myocardial vasodilatory response after cold test [7]. In the pathogenesis of myocardial damage, ischemia of small coronary arteries play a key role. We can suppose that myocardial ischemia can be responsible for DD and AD. The sST2 data, although with low significance and spread for the small sample size, could be useful in assessing myocardial damage. However, these preliminary data need to be confirmed in large studies before they can be used in clinical practice.

Our results demonstrated that a negative linear correlation exists between TAPSE/sPAP and sST2, conversely no correlation exists between TAPSE/sPAP and IL-33. A reduced TAPSE/sPAP ratio has been associated with poor outcomes in patients with pulmonary arterial hypertension (PAH). TAPSE/sPAP ratio is a powerful predictor of all-cause mortality in patients with PAH. In SSc patients, TAPSE/sPAP ratio can be used to further select patients requiring right heart catheterization to confirm PAH diagnosis and it showed the best correlation with ventilatory efficiency and exercise capacity [27, 28]. There are no data in the literature showing the role of sST2 in right ventricular dysfunction. We can suppose that sST2 can play a role in the pathogenesis of right ventricular–pulmonary arterial coupling, but our results cannot prove finalconclusions.

Main limitations of this study included the monocentric design, small sample size, unavailability of TTE or ECG data of healthy controls, unavailability of right hearth catheterization, the lack of myocardial histological findings.

The role of either these biomarkers in the fibrotic and vasculopathic manifestations of SSc is unknown from these study results, as only an associations were observed, and no assumption can be made about a pathogenic role for these biomarkers from this data.

In conclusion, serum levels of IL-33 and sST2 are increased in SSc patients. Serum levels of sST2 are a potential marker of DD, AD and right ventricular–pulmonary arterial coupling. Future larger studies are needed to confirm our preliminary data.
